# Carboxylated ε-Poly-l-lysine Improves Post-Thaw Quality, Mitochondrial Functions and Antioxidant Defense of Goat Cryopreserved Sperm

**DOI:** 10.3390/biology12020231

**Published:** 2023-02-01

**Authors:** Weijing Zhang, Haixiang Cui, Kexin Ding, Kaifeng Zhou, Yajing Li, S. A. Masudul Hoque, Lingjiang Min, Zhendong Zhu

**Affiliations:** 1 College of Animal Science and Technology, Qingdao Agricultural University, Qingdao 266109, China; 2 Shandong Provincial Animal Husbandry General Station, Jinan 250022, China; 3 Department of Animal Breeding and Genetics, Faculty of Veterinary Medicine and Animal Science, Bangabandhu Sheikh Mujibur Rahman Agricultural University, Gazipur 1706, Bangladesh

**Keywords:** goat sperm, cryopreservation, CPLL, quality

## Abstract

**Simple Summary:**

Goat sperm membrane is vulnerable to damage through ice crystal formation during cryopreservation. Recently, carboxylated ε-poly-l-lysine (CPLL) has been regarded as one of the novel cryoprotectants. This study was conducted to investigate the effect of CPLL on the post-thaw quality of goat cryopreserved sperm. It was observed that supplementation of CPLL to the cryopreservation medium enhances the motility, acrosome and membrane integrity, mitochondrial function, ATP production, and anti-oxidant defense mechanisms of post-thaw sperm while decreasing MDA level, total ROS production, and apoptosis. Therefore, CPLL can be supplemented during cryopreservation of goat sperm to maintain post-thaw quality and antioxidant activity.

**Abstract:**

Carboxylated ε-poly-l-lysine (CPLL), a novel cryoprotectant, can protect the sperm membranes by inhibiting ice crystal formation during the cryopreservation process. The present study was conducted to investigate the consequence of CPLL supplementation on the post-thaw quality of cryopreserved goat sperm. For this, different doses (0, 0.5%, 1%, 1.5%, and 2%; *v*/*v*) of CPLL were added to the cryopreservation medium, and the motility, membrane and acrosome integrity, mitochondrial membrane potential (MMP), ATP level, ROS production, anti-oxidant defense system, malondialdehyde (MDA) level, and apoptosis in post-thaw sperm were evaluated. It was observed that the addition of 1% CPLL significantly (*p* < 0.05) increased the total motility, membrane integrity, acrosome integrity, and catalase (CAT) activity of post-thaw sperm compared to those of control and other CPLL doses. The ATP content was observed significantly (*p* < 0.05) higher in 0.5% and 1% CPLL, however, the SOD activity and progressive motility were significantly (*p* < 0.05) increased by adding CPLL at 1% and 1.5% level. Moreover, the addition of CPLL at 1% dose not only showed a lower percentage of apoptosis, but also significantly (*p* < 0.05) increased the MMP while reducing ROS production and MDA levels compared to those of other CPLL doses and/or control. Therefore, it is clear that the supplementation of 1% CPLL can remarkably improve the post-thaw goat sperm motility, membrane and acrosome integrity, antioxidant abundance, mitochondrial potentials, and ATP supply by protecting the sperm from cryodamage and undergoing apoptosis. These findings will provide novel insights into sperm cryobiology.

## 1. Introduction

The use of high-quality frozen semen not only saves time, space, and life span but also can accelerate genetic progress by reducing the breeding cost [1,2]. However, the freezing–thawing process exerts irreversible impairment on sperm quality. These damages may come either through the alteration of osmotic pressure [3], reactive oxygen species (ROS) [4], or mechanical damage by ice crystal formation [5], which not only disrupts the plasma membrane but also causes homeostasis imbalance and leakage of antioxidant enzymes. Goat sperms are naturally sensitive to low temperature and osmotic stress damage. During the sperm cryopreservation process, part of the water outside the sperm membrane freezes first and then repels the solute to the unfrozen part forming a hypertonic solution. Additionally, the irregular shape of water-ice crystallization coupled with expanding ice crystals that are developed during freezing cause mechanical pressure on the sperm protoplasm layer and internal structure leading toward cryodamage [6]. Therefore, it is necessary to protect the sperm from cryoinjuries at the augmentation of freezing procedures.

During sperm cryopreservation, vitrification is most widely used in human [7] while slow freezing is popularly practiced in livestock animals [8,9]. It is reported that the vitrification process is superior to slow freezing considering the ice-crystallization along with the quality and fertilizing ability of post-thaw sperm [10]. However, the addition of an ideal cryoprotectant is necessary to prevent intracellular and extracellular ice-crystal formation in both slow freezing and vitrification processes. Recently, cryobiologists and reproductive biologists have been focusing on the use of potential additives to improve sperm quality and fertilization ability by protecting them from freezing damage. Cryoprotectants are important agents that protect the cells or tissues by preventing ice crystal formation from intracellular and/or intercellular water during the cryopreservation process. Among the nonpermeating cryoprotectants, Polyvinylpyrrolidone (PVP) and polyethylene glycol (PEG) are most extensively used. In addition, dimethyl sulfoxide (DMSO), glycerol, and ethylene glycol (EG) are used as permeating cryoprotectants for cryopreservation [11]. However, the use of these cryoprotectants may cause epigenetic modification or exert a toxic effect on gametes that lower fertilizing ability [12]. DMSO was observed to impair the epigenetic DNA methylation and histone profile of mouse embryos, and EG had a toxic effect on the vitrified mice unfertilized oocytes [13]. Working on fish spermatozoa, de Olena et. al. (2021) reported that the use of cryoprotective agents in sperm cryopreservation resulted in a significant alteration of DNA methylation pattern [14]. Moreover, use of DMSO exerts an antagonistic effect on the cryoprotectant ability of glycerol and reduces the post-thaw quality of buffalo sperm [15]. Studies on antioxidant supplementation in the cryopreservation process have suggested that the use of multiple antioxidant combinations may provide better protection against oxidative-induced damages than single antioxidants [16]. However, antioxidants alone cannot protect the sperm membrane from damage due to ice crystals [17]. Therefore, close attention is necessary to develop novel cryoprotective agents that have better efficiency and less toxicity than currently available cryoprotectants.

Antifreeze glycoproteins (AFGP) are reported to inhibit ice crystal growth and consequently, improve the progressive motility and plasma membrane integrity in buffalo bull sperm [18]. The CPLL has been effectively used as an anti-freeze molecule during the preservation of mesenchymal stem cells of rats and L929 cells of mice [19]. The ε-poly-l-lysine (ε-PL), a homo-polyamide biopolymer, consists of 25–35 l-lysine residues, ε-amino groups, and succinic anhydride that become ammonolysis to form CPLL [20]. CPLL has been commonly used in the fields of medicine, food, electronics, and clinical chemistry because of its special physical and chemical properties. Moreover, CPLL as an additional CPA has been used in the cryopreservation of various cell types, including the rat pancreatic islets [21], pig sperm [22], bovine somatic cells [23], mouse oocytes [24], human pluripotent stem cells [25], and natural killer cells [26]. Furthermore, CPLL showed a higher survival rate and in vitro developmental ability on Nili-Ravi buffalo sperm [27] and pig embryos [28]. However, no such study investigated the use of CPLL for sperm freezing in goats. Moreover, the mechanism of CPLL on the cryopreservation of sperm is still unknown. Therefore, the present study was conducted to investigate whether CPLL, as a novel cryoprotectant, has effects on the quality, mitochondrial activity, antioxidant defense mechanisms, and apoptosis of post-thaw goat sperm.

## 2. Materials and Methods

### 2.1. Chemicals 

All chemicals were obtained from Sigma-Aldrich (Shanghai, China) unless specified.

### 2.2. Semen Collection, Freezing, and Thawing 

Semen was collected from five healthy mature Laoshan bucks with an artificial vagina once per week according to Zhang et al. (2022a) [29]. Ejaculated semen with more than 85% motility was used in this study. During the freezing process, the pooled semen from each replicate was diluted with an extender containing 83 mM trisodium citrate, 250 mM Tris, 69 mM glucose, 20% (*v*/*v*) egg yolk, 5% (*v*/*v*) glycerol and different concentrations of CPLL (0%, 0.5%, 1%, 1.5% and 2% *v*/*v*, final concentration) at a concentration of 1.0 × 10^8^ sperm/mL. Then the semen was gradually cooled to 4 °C and equilibrated for 30 min. The semen samples were then loaded into 0.25 mL plastic straws and horizontally placed 10 cm above the liquid nitrogen (LN) for 10 min. After that, the straws were stored in LN until use. After one week or more, the straws were thawed at 37 °C for 30 s and examined.

### 2.3. Sperm Motility 

Sperm motility was evaluated with a computer-assisted sperm analysis (CASA) system according to Zhang et al. (2022b) [22]. Briefly, the sperm sample was dropped on a pre-warmed Leja 20 μm chamber and evaluated by CASA for total motility, progressive motility, VSL, VCL, VAP, LIN, WOB, ALH, STR, and BCF parameters. 

### 2.4. Membrane Integrity and Acrosome Integrity of Sperm

The membrane integrity of sperm was evaluated using SYBR-14 (Sigma-Aldrich, Shanghai, China) in combination with propidium iodide (PI) as described by Zhang et al. (2022a) [29]. Briefly, 100 µL of sperm sample was diluted with 100 µL TCG extender containing 0.1 µL SYBR-14 and 0.5 µL PI and then incubated in dark at 37 °C for 10 min. The TCG extender was composed of 83 mM trisodium citrate, 250 mM Tris, and 69 mM glucose. The stained sperm were evaluated with a fluorescence microscope (ZEISS DM200LED, Oberkochen, Germany). At minimum, 200 sperm per slide were examined and scored. 

The acrosome integrity of sperm was evaluated using fluorescence isothiocyanate-peanut agglutinin (FITC-PNA) and PI according to the previously developed protocol [29]. Briefly, 30 µL of sperm was smeared on the slide and fixed in 75% methanol. The fixed sperms were then incubated with 100 µg/mL FITC-PNA solution at 37 °C in the dark for 30 min, and further incubated with 2.4 mM PI stocking solution for 10 min. Acrosome integrity was observed with a fluorescence microscope (ZEISS DM200LED) with 488 nm excitation for FITC-PNA green fluorescence and 535 nm excitation for PI red fluorescence. At minimum, 200 sperm per slide were examined. Sperm displaying bright green were considered to have intact acrosome, whereas the absence of green or display of patchy green fluorescence was considered to have damaged acrosome.

### 2.5. Mitochondrial Membrane Potential (MMP) of Sperm

Sperm mitochondrial activity was evaluated using JC-1 Mitochondrial Membrane Potential Detection Kit (C2003S, Beyotime Institute of Biotechnology, Nantong, China) according to Zhu et al. (2019a) [30]. Only the best concentration of CPLL as obtained from the previous analyses was evaluated for MMP in this study. The aggregates in the mitochondrial matrix show red fluorescence in high MMP and green fluorescence in low MMP. For this purpose, 100 µL of sperm sample (5 × 10^6^) was stained with 10 µL JC-1 solution and incubated at 37 °C in the dark for 30 min. Then, samples were centrifuged at 600× *g* for 3 min at 4 °C. Subsequently, samples were washed twice and resuspended with JC-1 buffer on ice. After that, the sperm were filtrated with 40 µm cell strainers to separate the doublets. The staining sperm was taken on a glass slide and appraised with high mitochondrial membrane potentials in the FL3 channel (535 nm), and low mitochondrial membrane activity in the FL2 channel (488 nm) via a fluorescent microscope (ZEISS DM200LED). In addition, the staining samples were also evaluated using a flow cytometer (FACSAria III, BD Biosciences. Franklin Lakes, NJ, USA). A total of 20,000 sperm events were analyzed. 

### 2.6. ATP Levels

ATP levels of sperm were assessed with the ATP Assay Kit (A095-1-1, Nanjing Jiancheng Bioengineering Institute, Nanjing, China) according to the manufacturer’s instructions and previously developed protocol [31]. Briefly, semen samples (10^7^ sperm) were washed with ddH_2_O, resuspended, and boiled for 10 min. Then, 200 µL working solution was added to the samples and loaded onto a 96-well plate. Following 5 min incubation at room temperature, the absorbance was measured with a microplate reader (TECAN, Infinite M Nano, Männedorf, Switzerland) at 636 nm.

### 2.7. Detection of Intracellular Reactive Oxygen Species (ROS)

Intracellular reactive oxygen species (ROS) level was measured using a Reactive Oxygen Species Assay Kit (S0033M, Beyotime Institute of Biotechnology, Nantong, China) according to the manufacturer’s instructions and previously developed protocol [32]. Only the best concentration of CPLL as obtained from the previous analyses was evaluated for ROS. Briefly, 70 µL sperm samples were diluted with 150 µL TCG and centrifuged at 800× *g* for 5 min to wipe off egg yolk and glycerol. Then, resuspended with 200 µL Dichlorodihydrofluorescein diacetate (DCFH-DA) working solution, and incubated at 37 °C in the dark for 30 min. The level of DCF fluorescence generated from the oxidation of non-fluorescent DCFH was measured by a flow cytometer (FACSAria III, BD Biosciences). A total of 20,000 sperm events were analyzed.

### 2.8. Antioxidant and Enzyme Activity in the Thawed Sperm

Frozen-thawed sperm with diluter were lysed, thoroughly centrifuged for 5 min at 2000 rpm, and stored on ice until analysis. The concentration of total glutathione (GSH), malondialdehyde (MDA), catalase (CAT), and superoxide dismutase (SOD) levels were evaluated by commercial kits (Nanjing Jiancheng Bioengineering Institute, Nanjing, China) using a microplate reader (TECAN, Infinite M Nano, Männedorf). All analyses were executed based on the manufacturers’ instructions. For each parameter, the mixed reagent was transferred into a 96-well plate and evaluated according to the recommended absorption wavelengths. 

### 2.9. Western Blotting Analysis

Western blot analysis was done according to Zhu et al. (2019b) [33]. Briefly, the sperm total protein was extracted in sodium dodecyl sulfate (SDS) buffer and 20 µg protein from each sample was alienated by 10% SDS-PAGE gel (EC0023-B, Sparkjade, Jinan, China) and transferred against a polyvinylidene fluoride (PVDF) membrane. Blocking of non-specific bindings were done using 5% (m/v) BSA diluted with TBST (1% TBS, 0.1% Tween 20). After that, the membranes were immunoblotted with primary antibodies Caspase-3 (A2156), Caspase-9 (A2636), BCL-2 (A0208), Bax (A0207), and tubulin (AC008), diluted in TBST solution at 1:1000 ratio and incubated for 12 h at 4 °C. The membranes were then swept with TBST buffer and incubated in secondary antibodies (AS014) at 1:1000 ratio for 1 h. After three repeated washing with TBST, the blots were detected by ECL plus (ED0016-B, Sparkjade, Jinan, China) and developed by a gel imaging analyzer (Alpha, Fluor Chem Q, Shanghai, China). All the antibodies were purchased from AB clonal, Wuhan, China. 

### 2.10. Statistical Analysis

Values obtained upon 5 replicated assessments were analyzed with the Statistical Package for the Social Science (SPSS Statistics v26.0; IBM, Inc., Amunc, NY, USA). All results are presented as mean ± SEM. One-way ANOVA or *t*-test was performed for the level of significance considering the probability level of <0.05. 

## 3. Results

### 3.1. Effects of Carboxylated ε-Poly-l-lysine on Sperm Motility Parameters 

The motility patterns of sperm were evaluated by CASA and the results are summarized in Table 1. It was observed that the total motility and progressive motility were significantly (*p* > 0.05) increased in 1% CPLL supplemented sperm compared to those of control and other treatment (0.5%, 1.5%, and 2%) groups. The VSL, VCL, and VAP were observed as significantly (*p* > 0.05) higher in 0.5% to 1.5% CPLL supplemented groups compared to those of control and 2% CPLL group. However, 1% CPLL showed better results over other doses (Table 1). Although the LIN (%) was observed significantly increased by 0.5% and 1% CPLL supplementation, the WOB (%) was found significantly higher only in 1% CPLL supplemented sperm compared to those of control and other treatments. However, there was no significant (*p* > 0.05) difference observed in ALH, STR, and BCF parameters among the treatments. Considering all the parameters studied, it is clear that the addition of 1% CPLL to the freezing media could improve the motility parameters of post-thaw goat sperm. 

### 3.2. Effects of Carboxylated ε-Poly-l-lysine on Sperm Membrane and Acrosome Integrity 

**Figure 1:**
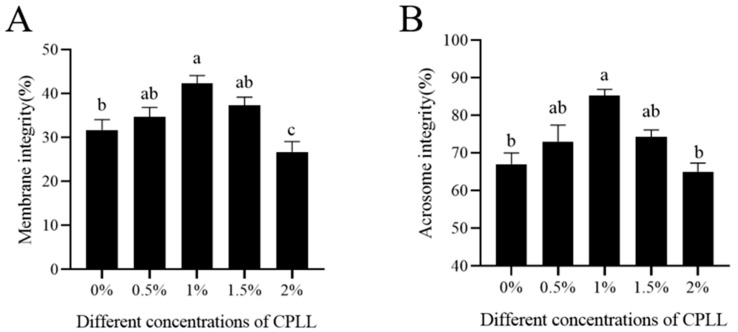
Effects of CPLL supplementation at 0% to 2.0% level to the freezing medium on post-thaw goat sperm membrane integrity (**A**) and acrosome integrity (**B**). Values are specified as mean ± standard deviation (SD). Columns with different lowercase letters differ significantly (*p* < 0.05).

As shown in Figure 1A,B, it was observed that supplementation of 1% CPLL to the goat sperm freezing media significantly (*p* < 0.05) increased the membrane integrity and acrosome integrity compared to the control and other treatments (0.5%, 1.5% and 2% CPLL). However, the addition of CPLL at 2% dose significantly decreases the membrane integrity compared to the control (Figure 1B). The results suggested that 1% CPLL supplementation was optimum to maintain intact membrane and acrosome during cryopreservation of goat sperm. 

### 3.3. Effects of Carboxylated ε-Poly-l-lysine on Mitochondrial Membrane Potential and ATP Level 

**Figure 2:**
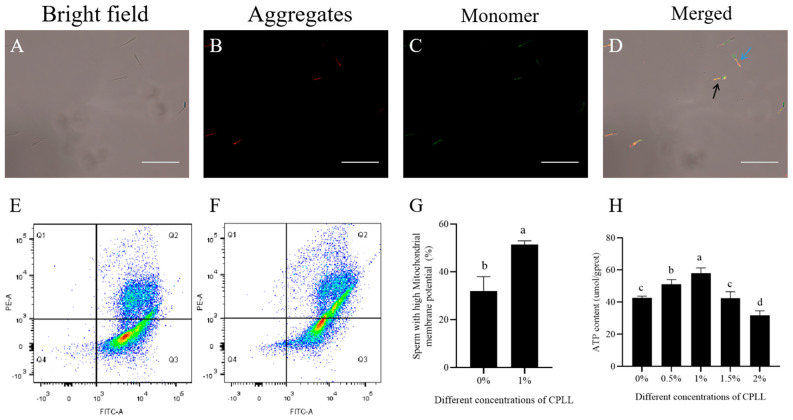
(**A**–**D**) Fluorescence microscopic observation of the mitochondrial membrane potential (MMP) of post-thaw goat sperm cryopreserved with or without CPLL, where the blue arrows indicate high MMP and the black arrows indicate low MMP. (**E**,**F**) MMP fluorescence emission in flow cytometry and (**G**) the comparison of MMP at 1% CPLL supplementation to the control. (**H**) Effects of CPLL supplementation at 0% to 2.0% level to the freezing medium on post-thaw goat sperm ATP content. Values are specified as mean ± standard deviation (SD). Columns with different lowercase letters differ significantly (*p* < 0.05). Scar bar = 30 μm.

Since the sperm with 1% CPLL treatment showed better membrane and acrosome integrity, the mitochondrial membrane potential (MMP) was evaluated in sperm with 1% CPLL supplementation only. The epifluorescence of MMP has been shown in Figure 2A–D, where red/orange and green fluorescence indicate high and low potential, respectively. The flow cytometry analysis of MMP has been shown in Figure 2E–G. As shown in Figure 2G, it was revealed that the MMP of frozen-thawed sperm was significantly (*p* < 0.05) increased with 1% CPLL treatment groups than those of control. 

ATP is essential for better maintenance of sperm motility and velocity. It was observed that the 0.5% and 1% CPLL supplementation significantly (*p* < 0.05) increased the ATP content of post-thaw sperm compared to the control (Figure 2H). However, similar ATP content was observed between the 1.5% and control group (*p* > 0.05), which was significantly (*p* < 0.05) decreased if supplementation of CPLL increased to 2% (Figure 2H). The results suggested that CPLL supplementation at 2% concentration is detrimental considering the energy supply to the sperm. 

### 3.4. Effects of Carboxylated ε-Poly-l-lysine on Sperm ROS Level and Malondialdehyde Content 

**Figure 3:**
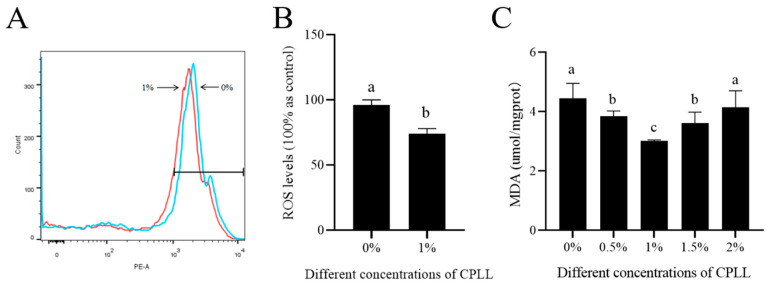
Effects of CPLL on goat sperm ROS content and MDA level after freezing-thawing. (**A**) Flow cytometer peak for ROS and (**B**) the ROS content compared between 1% CPLL and control. (**C**) The MDA level at different concentrations of CPLL supplemented to the freezing medium. Values are specified as mean ± standard deviation (SD). Columns with different lowercase letters differ significantly (*p <* 0.05). MDA: malondialdehyde; ROS: reactive oxygen species.

ROS generation in sperm was measured only in the best CPLL concentration (1%) group as observed in the previous analyses using flow cytometry and the results are shown in Figure 3A,B. In the results, the supplementation of 1% CPLL showed a significant (*p* < 0.05) reduction in the ROS content of frozen-thawed sperm compared to those of the control (Figure 3B). 

Additionally, malondialdehyde (MDA) level was analyzed and it was observed that the supplementation of CPLL from 0.5% to 1.5% CPLL treatments significantly (*p* < 0.05) reduced MDA content in frozen-thawed sperm, and 1% CPLL treatments showed the lowest malondialdehyde (MDA) levels compared to those of the control group (Figure 3C).

### 3.5. Effects of Carboxylated ε-Poly-l-lysine on Sperm GSH Levels, SOD and CAT Activity 

**Figure 4:**
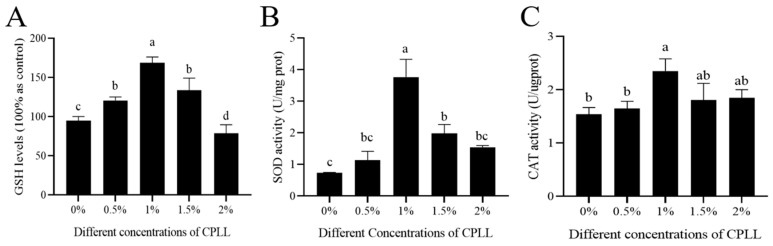
Effects of different concentrations of CPLL to the freezing medium on the antioxidant defense mechanisms of post-thaw goat sperm. (**A**) GSH levels, (**B**) SOD activity, and (**C**) CAT activity. GSH: Glutathione; SOD: superoxide dismutase; CAT: catalase. Values are specified as mean ± standard deviation (SD). Columns with different lowercase letters differ significantly (*p* < 0 05).

GSH levels, SOD activity, and CAT activity of post-thaw sperm were measured to evaluate the antioxidant potential of CPLL. It was observed that the supplementation of CPLL at 0.5% significantly (*p* < 0.05) increased the GSH level of post-thaw sperm, which was further significantly increased by increasing the level up to 1% level compared to the control. However, the GSH level was significantly (*p* < 0.05) decreased by further addition of CPLL to 1.5% and 2% levels compared to those of 1% CPLL (Figure 4A). The SOD activity was significantly (*p* < 0.05) increased by adding 1% and 1.5% CPLL to the freezing media compared to those of control, however 1% CPLL treatment showed almost five times higher SOD activity than those of the control group (3.75 ± 0.57 vs 0.74 ± 0.02, Figure 4B). The CAT activity of post-thaw sperm was significantly (*p* < 0.05) increased by only 1% CPLL supplementation, whereas the other levels of CPLL did not show any improvement compared to the control (Figure 4C). 

### 3.6. Effect of Carboxylated ε-Poly-l-lysine on Post-Thaw Sperm Apoptosis

**Figure 5:**
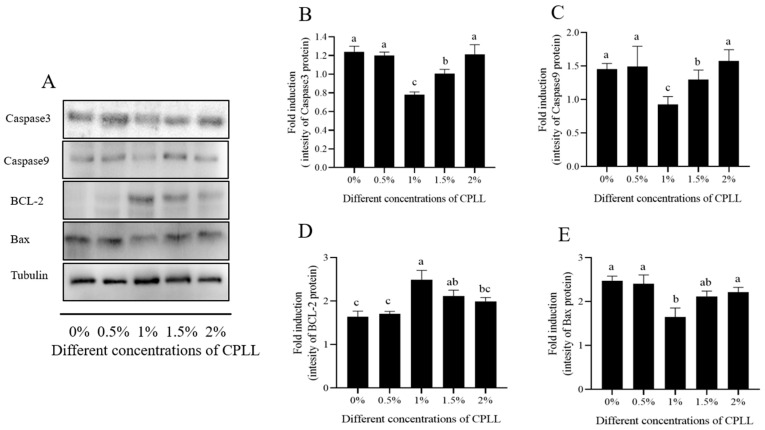
Detection of apoptosis-related proteins in post-thaw goat sperm by Western blotting. (**A**) The intensity of the bands analyzed using a Gel-Pro Analyzer (Media Cybernetics, MD, USA). (**B**–**E**) The quantitative expression of proteins over tubulin (internal control). Values are means ± standard deviation (SD) of 3 replicates. Columns with different lowercase letters differ significantly (*p* < 0.05).

Sperm apoptosis was detected in terms of Caspase-3, Caspase-9, BCL-2, and Bax by the western blot method with Tubulin as control (Figure 5A and Supplementary Figure S1). It was observed that the expression of Caspase-3 and Caspase-9 was significantly decreased in the 1% and 1.5% CPLL groups compared to other CPLL (0.5% and 2.0%) and control groups (Figure 5B,C; *p* < 0.05). The addition of 1% and 1.5% CPLL to the extender significantly (*p* < 0.05) increased the expression of BCL-2 in the frozen-thawed sperm; however, no improvement (*p* > 0.05) was observed in 0.5% and 2.0% CPLL groups compared to those of the control (Figure 5D). The level of Bax was significantly (*p* < 0.05) decreased by 1.0% CPLL supplementation to the freezing extender; however, the other treatments (0.5%, 1.5%, and 2.0%) showed a similar (*p* > 0.05) expression as compared to those of the control. 

## 4. Discussion 

Cryopreservation of sperm is a sophisticated and thermo-sensitive procedure. The improper handling time and temperature during slow-cooling at low temperature leads to rapid-cooling injury [34]. Furthermore, the ice crystals formed during the freezing process cause mechanical damage to the sperm membrane and consequently, the leakage of essential enzymes [35]. Therefore, the use of cryoprotectants in the freezing medium is essential to protect the sperm from cooling injury. There are a variety of nonpermeating and permeating cryoprotectants used for the cryopreservation of cells and tissues; however, their use in the haploid gamete may cause epigenetic modification or exert a toxic effect [13]. In recent years, carboxylated poly-l-lysine (CPLL) has been developed as a new nonpermeable cryoprotectant composed of the ampholytic polymer of ε-poly-l-lysine (ε-PL) and succinic anhydride.

CPLL is a complex molecule, hexagonal or bipyramid in shape which can change the morphology of large ice crystals and remove small ice crystals. In addition, polycationic molecules in poly-lysine solution and glycoproteins molecules of CPLL are easy to adsorb on the hydrophilic surface of the cells. Therefore, a negative pressure is created inside that exudates the intra-cellular moisture outward and reduces the ice crystal formation, and protects the cell from membrane damage [36,37]. Zhang et al. (2022a) [22] reported that freezing injury in boar sperm could be diminished by supplementation of 0.25% CPLL into a 1% glycerin-based cryoprotective solution when they checked the CPLL concentration from 0% to 1%. The different dose effects of CPLL on sperm cryopreservation between the boar and goat species may be due to the different freezing procedures and medium composition. Hiroki Takeuchi et al. (2021) [25] reported that cryopreservation reagents containing CPLL, glycerol, and raffinose showed similar results in terms of viability, motility, and DNA fragmentation in human sperm. These studies suggested that the optimal concentration of CPLL can be used as cryoprotective agents for cryopreservation of sperm. In a previous study, we used CPLL as a cryoprotectant during boar sperm cryopreservation [22]. In this study, we focused on the effects of CPLL on the cryopreservation of goat sperm and explored its optimal concentration. In this study, it was revealed that supplementation of 1% CPLL to sperm freezing media showed the best results considering motility, membrane and acrosome integrity, mitochondrial activity, and anti-oxidative capacity of post-thaw goat sperm. 

Mitochondria are powerhouses of energy for the motility and regulate autophagy, apoptosis, free radical production, and lipid metabolism with other physiological functions in sperm. Moreover, mitochondrial biogenesis is dependent on MMP and plays an imperative role in sperm quality. Thus, improving sperm mitochondrial function would enhance post-thaw sperm quality. In this study, the MMP and ATP production were significantly enhanced by the addition of 1% CPLL. In accordance with the present study, Zhang et al. (2022a) [22] found that CPLL treatment in the boar sperm dramatically improved the mitochondrial membrane potential. 

The antioxidant enzyme system in the testis is the main force to maintain the normal ROS level of sperm cell [38]. After ejaculation, the spermatozoa are subjected to the shortfall of antioxidants and are damaged by oxidative stress in the external environment. Consequently, the ROS level keeps rising and the oxidation rate exceeds the clearance rate resulting in the imbalance of the redox system [39]. Moreover, ROS oxidize the biofilms and form the lipid peroxide MDA. In our study, 1% CPLL supplementation to the goat sperm cryopreservation media not only significantly decreased ROS production and MDA levels but also significantly increased GSH levels and antioxidant enzymes (SOD and CAT) activity. The enhanced antioxidant activities and increased GSH level signify the role of CPLL in protecting membrane integrity and mitigating enzyme leakage towards the reduction of ROS and MDA. Therefore, it is clear that the supplementation of CPLL to the cryopreservation medium can protect and enhance the antioxidant defense mechanisms of the post-thaw goat sperm. These results agreed with the previous study findings in Nili Ravi buffalo sperm that CPLL supplementation decreased the MDA level [27].

It is well recognized that the survival of spermatozoa is mainly powered by mitochondrial ATP produced through oxidative phosphorylation in the electron transport chain complexes. Ice crystals not only damage the extracellular membrane but also disrupt the mitochondrial membrane and respiratory chain, leading to the leakage of cytochrome C, and the reduction of MMP. The decline of mitochondrial transmembrane potential is considered to be the earliest event in the apoptotic cascade [40]. Cytochrome C joins with Apaf-1 forming apoptosome and activates caspase-9. Then, caspase-9 elicits the caspase cascade resulting in the activation of caspase-3 and consequently leading to cellular apoptosis [41]. Moreover, loss of antioxidant enzymes intensifies the incidence of oxidative stress that lowers mitochondrial membrane potential causing an imbalance expression of the BCL-2 family including up-regulated pro-apoptotic (Bax) and down-regulated anti-apoptotic (BCL-2) factors [42]. In this study, the CPLL supplementation to the cryopreservation medium not only improved the acrosomal and membrane integrities but also decreased apoptosis of post-thaw goat sperm.

## 5. Conclusions 

The present study showed that the supplementation of CPLL as a cryoprotective agent to the freezing medium can enhance the motility, acrosome integrity, and membrane integrity together with an increase in ATP content, mitochondrial function, and anti-oxidant defense system while decreasing MDA content, ROS production, and apoptosis in goat post-thaw sperm. Considering the comparative results among the doses, 1% CPLL can be used as an effective cryoprotectant in goat sperm cryopreservation.

## Figures and Tables

**Table 1:** Effect of CPLL on post-thaw goat sperm motility parameters measured with CASA.

Sperm Parameters	0%	0.5%	1%	1.5%	2%
Total motility (%)	35.85 ± 3.21 ^b^	39.02 ± 3.95 ^b^	49.33 ± 2.61 ^a^	42.13 ± 3.67 ^b^	37.00 ± 2.48 ^b^
Progressive motility (%)	24.5 ± 1.72 ^b^	26.98 ± 1.12 ^b^	35.53 ± 1.54 ^a^	27.5 ± 1.73 ^b^	23.50 ± 1.81 ^b^
VCL (μm/s)	34.37 ± 5.48 ^b^	46.18 ± 3.16 ^a^	53.8 ± 3.95 ^a^	47.9 ± 2.37 ^a^	33.46 ± 1.67 ^b^
VSL (μm/s)	13.45 ± 2.03 ^c^	24.64 ± 2.14 ^b^	35.94 ± 4.50 ^a^	24.85 ± 1.25 ^b^	16.06 ± 0.7 ^c^
VAP (μm/s)	18.49 ± 3.82 ^c^	30.09 ± 2.78 ^b^	40.42 ± 4.13 ^a^	30.38 ± 1.25 ^b^	20.84 ± 0.87 ^c^
BCF (Hz)	8.48 ± 0.58	8.18 ± 1.47	7.57 ± 1.18	9.03 ± 0.46	9.23 ± 1.01
ALH (μm)	2.99 ± 0.32	3.03 ± 0.22	2.88 ± 0.42	2.77 ± 0.1	2.88 ± 0.08
STR (%)	78.25 ± 1.05	77.9 ± 2.36	84.76 ± 3.93	84.18 ± 1.52	79.03 ± 1.86
LIN (%)	47.79 ± 1.19 ^b^	57.11 ± 14.96 ^a^	57.86 ± 0.73 ^a^	52.85 ± 3.06 ^ab^	46.23 ± 1.27 ^b^
WOB (%)	61.08 ± 1.43 ^b^	65.52 ± 3.36 ^b^	74.10 ± 1.96 ^a^	65.82 ± 2.41 ^b^	66.12 ± 2.46 ^b^

Values are expressed as mean ± standard deviation. Different letters within the same row differ significantly (*p* < 0.05). VCL, curvilinear velocity; VSL, straight-line velocity; VAP, average path velocity; BCF, beat-cross frequency; ALH, lateral head; STR, straightness (VSL/VAP); LIN, linearity (VSL/VCL); WOB, wobble (VAP/VCL), *n* = 5.

## Data Availability

Not applicable.

## Supplementary Materials

The following supporting information can be downloaded at: https://www.mdpi.com/article/10.3390/biology12020231/s1, Figure S1: Effects of different concentration of carboxylated ε-poly-l-lysine on protein (Caspase 3, Caspase 9, BCL-2 and Bax) expression in post-thaw goat sperm.
