# Supplementary material for: Carboxylated ε-Poly-l-lysine Improves Post-Thaw Quality, Mitochondrial Functions and Antioxidant Defense of Goat Cryopreserved Sperm
Source: Biology (Basel). 2023 Feb 1;12(2):231. doi: 10.3390/biology12020231 (PMC9953348; doi:10.3390/biology12020231)
Supplement: Supplementary file 1 [file biology-12-00231-s001.zip › biology-2113960-supplementary.pdf]

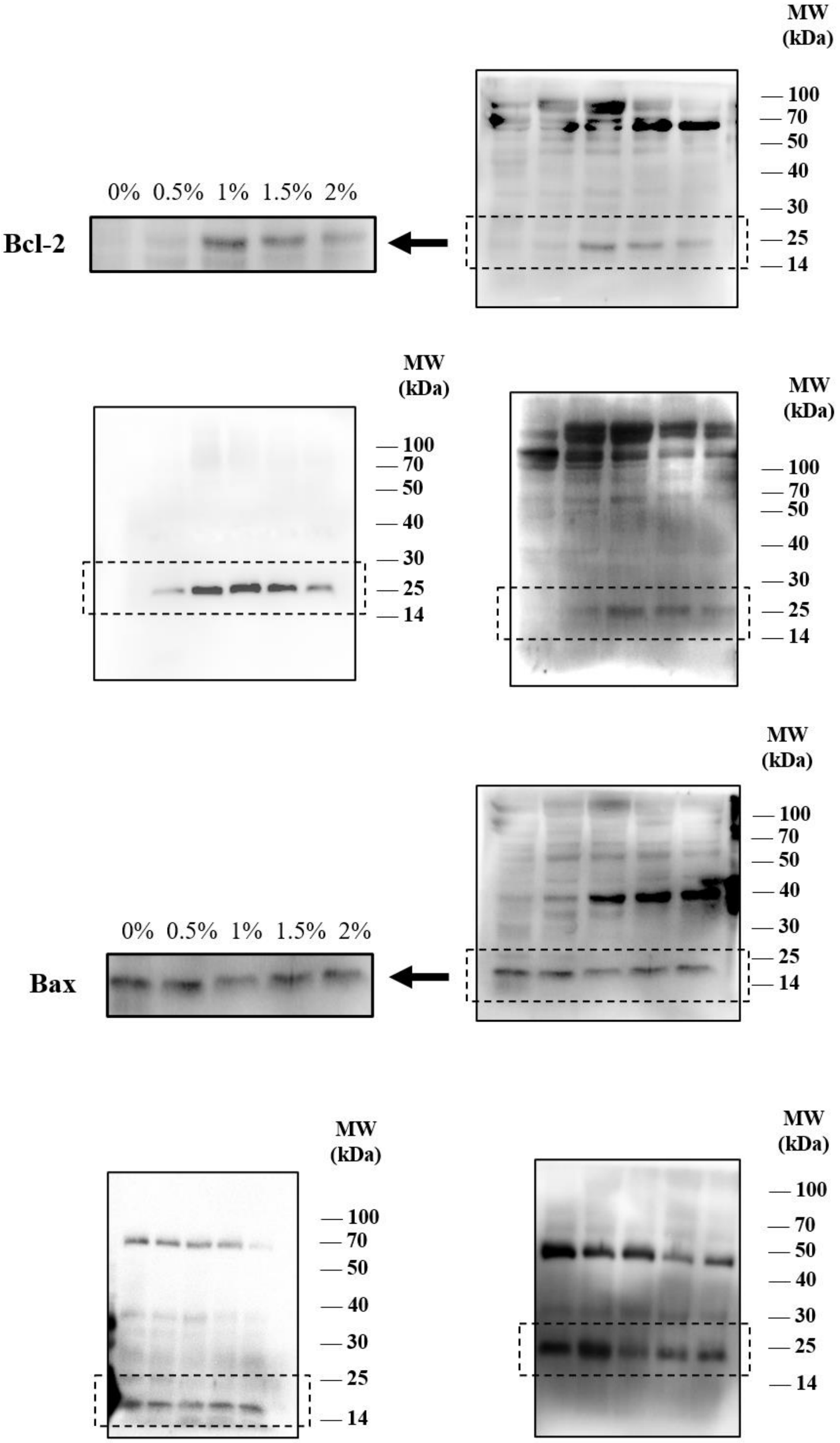

0% 0.5% 1% 1.5% 2%
Bcl-2
MW (kDa)
100
70
50
40
30
25
14
0% 0.5% 1% 1.5% 2%
Bax

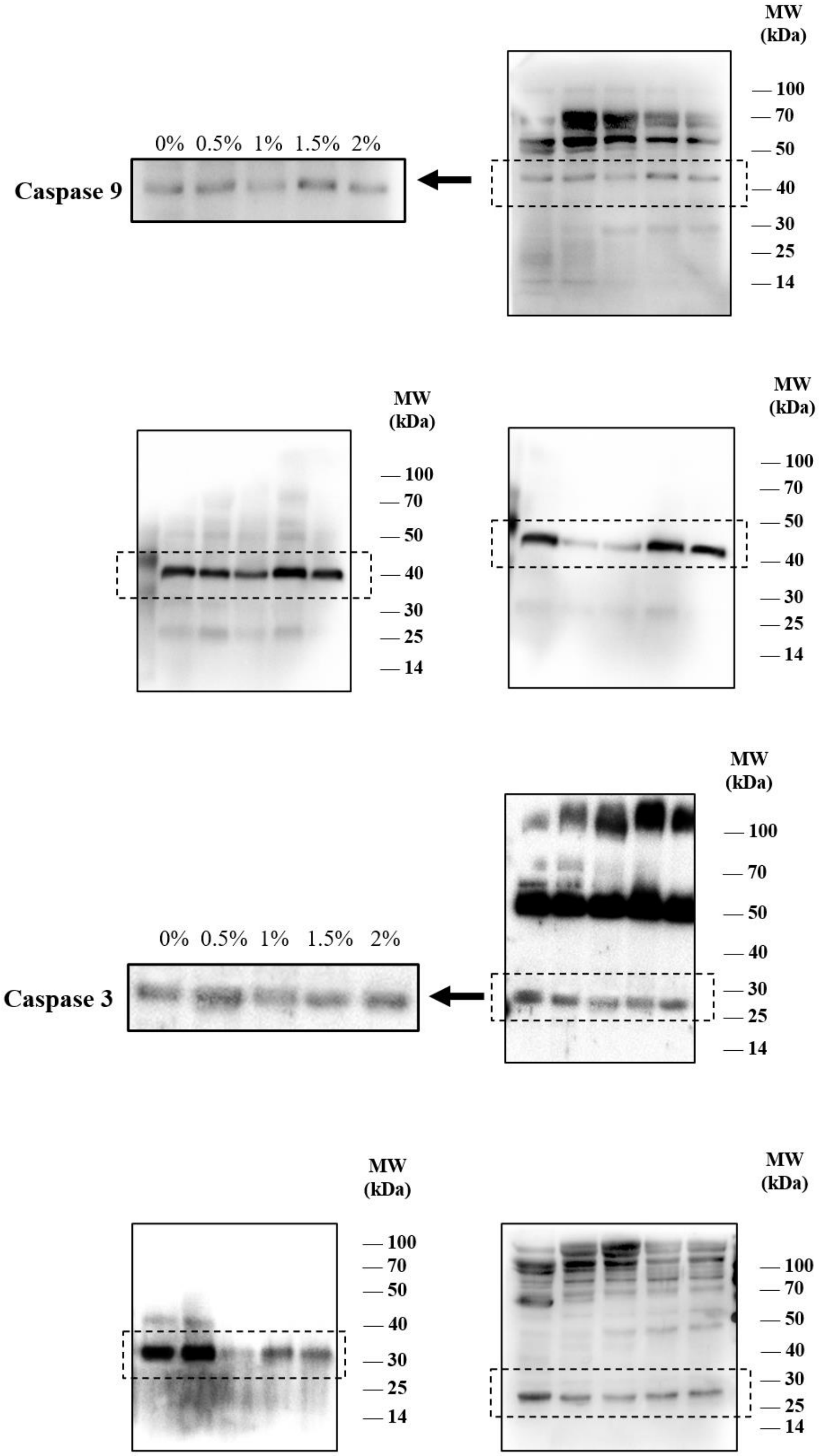
Caspase 9
0% 0.5% 1% 1.5% 2%
MW (kDa)
100
70
50
40
30
25
14
Caspase 3
0% 0.5% 1% 1.5% 2%

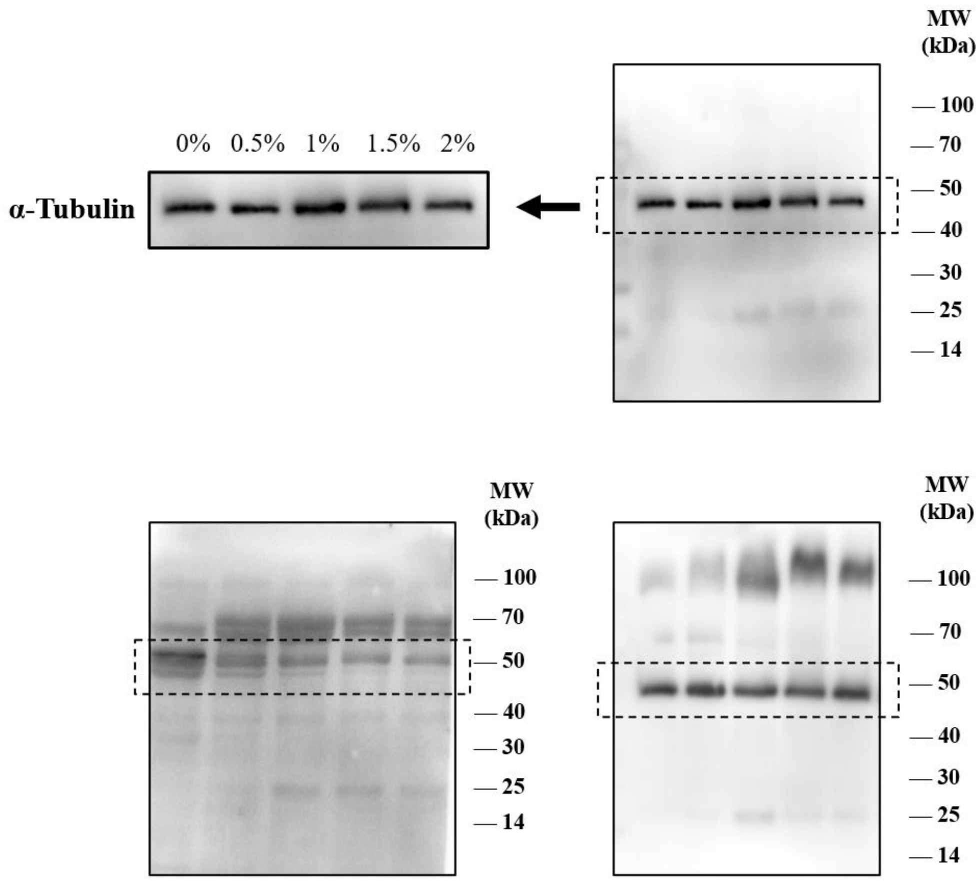

Figure S1. Effects of different concentration of carboxylated ε-poly-L-lysine on protein (Caspase 3, Caspase 9, Bcl-2 and Bax) expression in post-thaw goat sperm.
